# A Low-Cost Filament Winding Technology for University Laboratories and Startups

**DOI:** 10.3390/polym14051066

**Published:** 2022-03-07

**Authors:** Artem Andrianov, Erika Kamada Tomita, Carlos Alberto Gurgel Veras, Bruno Telles

**Affiliations:** 1 Aerospace Engineering Course, University of Brasilia, Área Especial de Indústria Projeção A, Setor Leste (Gama), Brasília 72444-240, Brazil; erika.kamadat@gmail.com (E.K.T.); bruno@telles.com.br (B.T.); 2 Mechanical Engineering Department, Campus Universitário Darcy Ribeiro, University of Brasilia, Brasília 70970-900, Brazil; gurgel@unb.br

**Keywords:** filament winding, winding trajectory, composite casing, polymer composite

## Abstract

This paper systematically explains the methodology and results of empirical work on the development of a low-cost filament winding technology for manufacturing axisymmetric polymer composite structures with a high length-to-diameter ratio, such as tubes, motor casings, and pressure vessels. The principal objective was to examine the experiences and most optimal practices in the development of computer-controlled equipment and auxiliary tooling for the wet filament-winding process. To preclude expensive commercial software for the automated control of a winding machine, analytical equations were derived for the winding trajectory of a four-axis filament-winding machine. The feasibility of the proposed equations was successfully validated by laying the fiber along the geodesic path marked on the surface of a cylindrical mandrel with hemispherical ends. Moreover, the carbon/epoxy cylindrical casings with hemispherical ends and port openings of the same diameter were wound to determine the thickness distribution in the hemispherical dome. The fiber volume ratio in the wound composite parts was evaluated using an optical technique.

## 1. Introduction

Filament winding technology has been deployed extensively in the aerospace industry since the early 1940s [1] and has persistently garnered interest from the scientific community. Data from Google Scholar, published in an insightful review on the automated manufacturing and processing of fiber-reinforced polymer composites, revealed the presence of a markedly high number of papers concerned with filament winding even in recent times [2]. However, the acquisition of composite structures with a unique shape of revolution and dimensions by university laboratories or startups requires sizeable investments. Under these conditions, small companies typically seek alternative solutions. University teams, however, can potentially benefit from sponsorship by equipment manufacturers, as demonstrated in the case of the production of filament-wound rocket fuselages [3]. Another tenable solution is the development of in-house low-cost equipment for filament winding [4].

A multitude of reports have highlighted the practicability of in-house laboratory equipment for assessing new filament winding techniques and for the experimental fabrication of advanced composite materials. Reference [5] presents a five-axis filament winding machine (FWM), including a numerical control unit and liner. It is suggested in [6] to increase the fiber volume ratio and decrease the void volume ratio of the composite casing. An environmentally friendly FWM, without any discernible loss of quality was devised by the separation of the resin components with their controllable feeding initially to a conventional static-mixer and subsequently to a custom-designed resin impregnation unit [7,8]. In the study reported in [9], the development of a controller using the B-spline interpolation technique entailed the construction of a three-axis FWM. A succinct description of the filament winding hardware that features precision guidance of the carbon nanotube-based material is given in [10]. A “spiral-winder” was developed for the manufacturing cylindrical laminated veneer lumber in [11]. The majority of the said research had a narrow purview with their focus on specific scientific problems and engineering solutions related to filament winding technology, and only a few details were presented regarding the cost-effective design of the filament winder, its control system, and winding technique.

The volume ratio and decrease in the void volume ratio of the composite casing were recorded. An environmentally friendly FWM, without any discernible loss of quality was devised by the separation of the resin components with their controllable feeding initially to a conventional static-mixer and subsequently to a custom-designed resin impregnation unit [7,8]. In the study reported in [9], the development of a controller using B-spline interpolation technique entailed the construction of a three-axis FWM. A succinct description of the filament winding hardware that features precision guidance of the carbon nanotube-based material is given in [10]. A “spiral-winder” was developed for the manufacturing cylindrical laminated veneer lumber in [11]. The majority of the said research works had a narrow purview with their focus on specific scientific problems and engineering solutions related to filament winding technology, and only a few details were presented regarding the cost-effective design of the filament winder, its control system, and winding technique.

The methodology and results of one of the first attempts to design a low-cost computer-operated wet filament winding system for manufacturing cylindrical and conical parts or a combination of components are delineated in [12]. The associated system is accompanied by a software written in the AUTOLISP parametric programming language for the visualization of the winding patterns. Another innovative lathe-type machine based on a wet winding method is presented in [13]. The cost of the system is low because of the implementation of a rigid automation: the control system is not controlled by a computer, but is rather based on relays, limit switches, a timer, and a counter. Similar low-cost design solutions have been presented, which are based on a speed control of two DC [14] or AC motors [15]. An ingenious small-scale FWM has also been developed for educational purposes [16]. This machine generates helical winding patterns with angles ranging from 40 degrees to 80 degrees. The mandrel is driven by a cheap AC motor with a relatively constant speed. The translational motion of the carriage with delivery eye is provided by a stepper motor, which is more sophisticated in terms of control. All of the mentioned systems have only two controllable axes that apply known limitations on a variety of wound parts.

An example of modernization of a two-axis winder by adding two more controllable axes is systematically detailed in [17]. In fact, one axis is adjusted manually (the distance between delivery eye and mandrill) and the other axes are driven by three DC motors with the same number of controllers. An efficacious low-cost solution is presented in [18], wherein a three-axis filament winder is equipped with one AC motor for the mandrel rotation and two AC servo-motors for the translation of the carriage. Servo-motors are controlled by the LabView software. A three-axis portable FWM was developed with an extensive use of the standard accessories for fabrication of hobby and laboratory machines, such as OpenBuilds V-Slot aluminum profile and other parts (bearings, fixtures, pulleys, belts, etc.) [19]. All the three axes of the winder are driven by stepper motors and controlled by the microcontroller Arduino Uno and drive the expansion board computer numeric control (CNC) Shield. Universal G-Code Sender software is used to send commands from the computer to the winder in [20]. More recently, winders were equipped with mobile software for wireless control [21]. The winding performance was assessed as satisfactory in accordance with the criteria of the angle deviations and accuracy of the distance between two adjacent helical roving positions in [22]. In addition, tubular structures have been manufactured with the use of commercial software for generation of winding trajectory [23]. Notwithstanding the low-cost design philosophy deemed critical in the aforementioned projects, the validation of the final product has been demonstrated for the structural components with a non-sophisticated shape, such as tubes.

A methodical description of a three-axis filament winder with an original technical solution for the third degree of freedom (DOF) is given in the references [24,25]. Fibers are consolidated by a twisting of the tow to form a bar of a circular cross-section. The twisting can be performed by a rotation of the delivery carriage. Nevertheless, the developed equipment only allows for the manufacturing of wrapped tow reinforced trusses. A meticulous description of small-scale equipment with four DOF that combines the filament winding with automated fiber placement is provided in reference [26]. A noteworthy advantage of the contemporary technology is the likelihood of manufacturing components with concave surfaces. However, the validation was fulfilled only with numerical simulations and not by manufacturing the test specimen, the former being less complex and arduous than the latter.

At present, there exist several commercial programs for generating the filament winding trajectories that use the mandrel’s shape as the initial data [27]. As stated in [28], a more intricate approach for path generation is currently under investigation to enhance the quality of the final product. The approach considers the alteration in the mandrel’s shape due to an uneven thickness distribution of the ply from previous winding. Acquisition of the commercial software for path generation increases the final cost of the technology, and the latter approach requires the application of expensive equipment.

Another way to make the technology more cost-effective is the development of analytical solutions to determine the winding trajectories performed by components of the FWM [29]. Depending on the shape of the mandrel, the fibers are laid along geodesic or non-geodesic trajectories. In accordance with reference [30], which contains analytical solutions for the winding paths of the parameterized shells of revolution, geodesic trajectories provide the most stable and economical technique for filament-wound structures.

An efficacious, viable solution for the smooth winding motion is delineated in [29]. However, it is applicable only for two-axis FWMs. An analytical solution for four- and five-axis machines is described in the form of the coordinates for the feed-eye trajectory and the mandrel rotation [5,31]. Nevertheless, the solution in reference [5] only provides satisfactory results for the spherical dome when the distance from the delivery eye to the surface of the mandrel is zero. A generic kinematic model of the machine movements to create a particular filament-wound product, on a particular machine configuration, is discussed in reference [32]. The mathematical approach adopted for the derivation of the executive expressions in [31,32] demands tremendous effort or skill from an FWM operator.

The primary objective of this work was to design low-cost computer-controlled equipment for the filament winding of small-size axisymmetric aerospace structures, such as tubes, casings, and pressure vessels. The secondary objective of the work was to obtain simple and comprehensive analytical equations for the delivery eye trajectory by referring only to analytic geometry. The derived equations were used for the winding of a cylindrical casing with hemispherical domes, whose polar openings are of the same diameter.

The validation of the suggested low-cost winding technology was conducted by manufacturing small-scale composite casings to evaluate the thickness and fiber volume ratio of helical plies. The dimensions of the designed casing are close to the size of the test motor of a hybrid propellant decelerator [33].

## 2. Materials and Methods

### 2.1. Constituents of the Composite Material

Carbon fiber, being the most difficult fiber to manage [1], has been used in this study in the filament-winding process to appraise the capability of the proposed system under demanding situations. Continuous carbon fiber tows based on polyacrylonitrile Teijin Carbon HTS45 E23 12K (Table 1) and epoxy resins of different grades produced by Huntsman (Table 2) were used for manufacturing the casing on the developed winding machine.

The fiber volume ratio was determined using an optical technique based on an image analysis of micrographs of transverse cross sections of the coupons. The fiber volume ratio was calculated as a ratio of the area of the fiber cross sections to the total area within the frame of the micrograph. The thresholding tool of the open-source software ImageJ 1.53e was used to determine the area of fiber cross sections on the 8-bit micrograph with a magnification of 400×.

### 2.2. Calculation of the Winding Parameters

**Figure 1:**
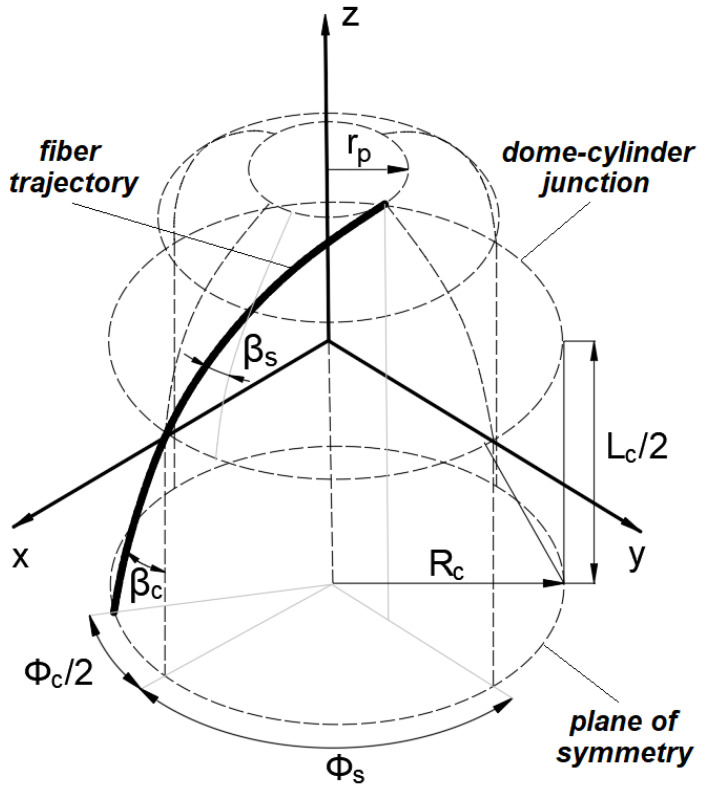
The definition of the winding parameters for a geodesic trajectory.

The winding parameters for the casing with hemispherical domes were calculated in accordance with the methodology and the recommendations delineated in [34,35]. The geodesic trajectory is feasible for the cylindrical pressure vessel with hemispherical domes of the radius *R_c_* and polar openings of the equal radius *r_p_* (Figure 1).

The winding angle for the cylinder $\beta_{c}$ was calculated by equation:

(1) $$\beta_{c}=\arcsin\frac{r_{p}}{R_{c}}$$

Along the length of the cylinder *L_c_*, the mandrel has to rotate with a turn-around angle $\Phi_{c}$ computed as follows:

(2) $$\Phi_{c}=\frac{L_{c}\tan\beta_{c}}{R_{c}}$$

The winding angle at any point on the surface of the hemispherical dome $\beta_{s}$ can be defined as a function of the z-coordinate as:

(3) $$\beta_{s}\left(z\right)\;=\arcsin\sqrt{\frac{R_{c}^{2}-z_{p}^{2}}{R_{c}^{2}-z^{2}}}$$

In addition, the turn-around angle on the hemispherical dome $\Phi_{s}$ can be defined as a function of the z-coordinate as well:

(4) $$\Phi_{s}\left(z\right)\;=\int_{0}^{z}\frac{1}{R_{c}^{2}-z^{2}}\sqrt{\frac{R_{c}^{2}-z_{p}^{2}}{z_{p}^{2}-z^{2}}}dz$$

To lay the fiber along one side of the hemispherical surface, the mandrel ought to rotate by a turn-around angle equal to $\Phi_{s}$, given as:

(5) $$\Phi_{s}=\int_{0}^{z_{p}}\frac{1}{R_{c}^{2}-z^{2}}\sqrt{\frac{R_{c}^{2}-z_{p}^{2}}{z_{p}^{2}-z^{2}}}dz=\frac{\pi }{2}$$

The turn-around angle for one winding cycle $\Phi_{1}$ can be computed as:

(6) $$\Phi_{1}=2\Phi_{c}+4\Phi_{s}$$

The fiber crosses any latitude of the mandrel twice every winding cycle. For the obtained value $\Phi_{1}$ not a multiple of 2*π* (360°), the tow after one winding cycle returns to the same latitude from which it started its trajectory but does not coincide with the starting point. The angular pitch of winding $\Phi_{p}^{*}$ is defined as an angle measured in the direction of the mandrel rotation between the starting and final points of one winding cycle and can be described mathematically as:

(7) $$\Phi_{p}^{*}=\Phi_{1}-2\pi \;\cdot \;integer\left(\frac{\Phi_{1}}{2\pi }\right)$$

The angular pitch must be increased to the closest angle, which is a multiple of 360°

$$\Phi_{p}=\;\left\{\begin{array}{c} 60{}^{\circ},\;72{}^{\circ},\;90{}^{\circ},\;120{}^{\circ},\;180{}^{\circ}\;when\;60{}^{\circ}\leq \Phi_{p}^{*}<180{}^{\circ}\\ 240{}^{\circ},\;270{}^{\circ},\;288{}^{\circ},\;300{}^{\circ},\;360{}^{\circ}\;when\;\Phi_{p}^{*}>180{}^{\circ} \end{array}\right.$$

The difference between the accepted and calculated angular pitch, $\Phi_{f}$, distributed uniformly between two flanges of the mandrel, can be given as:

(8) $$\Phi_{f}=\frac{\Phi_{p}-\Phi_{p}^{*}}{2}$$

At an angular pitch of less than or equal to 180°, the tow returns to the same starting point after $2\pi /\Phi_{p}$ winding cycles. Thus, to cover the entire surface of the mandrel with fibers, the mandrel must rotate every $2\pi /\Phi_{p}$ winding cycles by an angle corresponding to the width of the tow given as follows:

(9) $$\Phi_{w}=\frac{b}{R_{c}\cos\beta_{c}}$$

### 2.3. Validation of the Analytical Solutions for the Winding Trajectory

**Figure 2:**
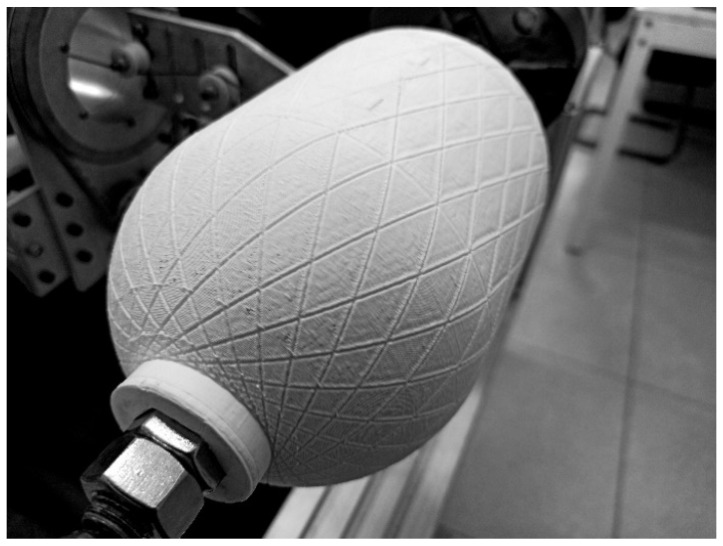
The mandrel with imprinted geodesic path.

The efficiency of the analytical solution for the kinematic motion of the FWM was ascertained by laying the synthetic strip along the geodesic path marked on the surface of a cylindrical mandrel with hemispherical domes. The mandrel with continuous grooves along geodesic path is printed with polylactide filament (Figure 2). The coordinates of the geodesic path were calculated by equations from the previous subsection. The G-code for controlling the four axes of the FWM was compiled manually after the discretization of the derived analytical solutions.

### 2.4. Manufacturing and Characterization of the Casing

**Figure 3:**
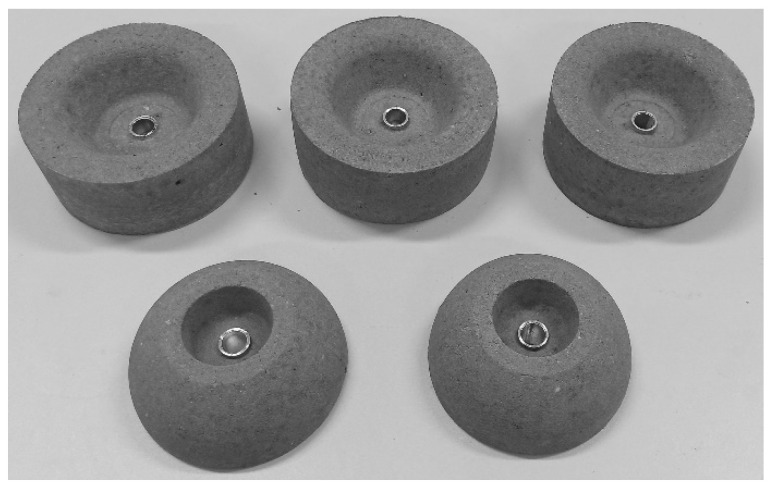
Solidified sand components kit for manufacturing a soluble mandrel.

**Figure 4:**
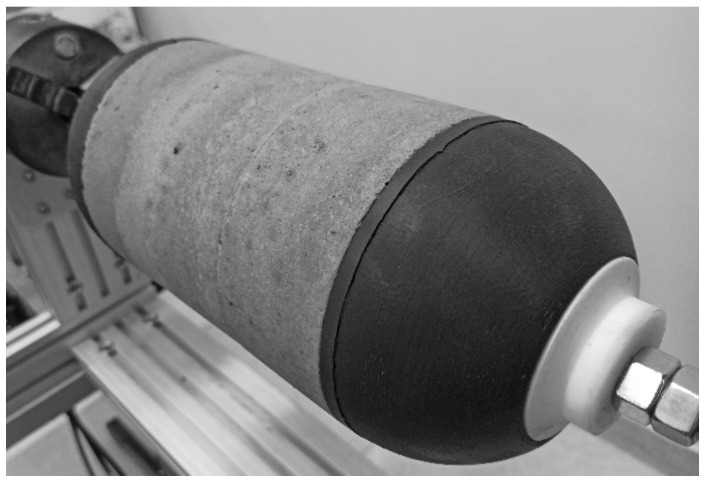
The sand mandrel assembled on a threaded shaft with flanges and heat insulators.

The casing is wound over two types of mandrel with the same geometry (*R_c_* = 60 mm, *r_p_* = 21 mm, and *L_c_* = 160 mm). The mandrel for the cold-curing epoxy composition LY5052 is made from polyethylene terephthalate glycolmodified (PETG) filament by 3D printing. Water-soluble mandrel from sand/polyvinyl alcohol composition [35] was used for the heat-curing epoxy resin LY1564. The sand components of the mandrel (Figure 3) were molded together with aluminum bushings in low-cost silicon molds. After solidification in an electric oven, these were mounted on a threaded shaft (Figure 4) with other components of the mandrel, namely heat insulators and flanges. The sand mandrel was washed out with hot water after manufacturing and curing the casing.

The average velocity of the winding was 65 mm/s and the winding pitch was 4 mm.

The thickness distribution for the spherical dome of the casing was evaluated by a so-called flat solution, suitable for a reliable and adequate approximation of real thickness distribution [1]. The thickness at the portion of the dome adjacent to the polar openings *t_do_* is constant and can be defined as:

(10) $$t_{do}=\frac{R_{c}t_{c}\cos\beta_{s}}{\sqrt{b\cos\left(\beta_{c}+\frac{b}{R_{c}}\right)\left[2r_{p}+b\cos\left(\beta_{c}+\frac{b}{R_{c}}\right)\right]}}$$

For the rest of the dome, the thickness *t_d_* is a function of the z-coordinate:

(11) $$t_{d}\left(z\right)\;=\frac{R_{c}r_{p}\cos\beta_{s}}{\sqrt{R_{c}^{2}-r_{p}^{2}-z^{2}}}$$

The predicted thicknesses were compared with the real ones measured on segments milled from the spherical dome of the wound casings. The measurements of the thickness were performed using a Mitutoyo digital caliper (resolution 0.01 mm) with thin jaws to avoid a distortion of the measured value due to the curvatures of the segments.

## 3. Results and Discussion

### 3.1. Design of the Filament Winder

#### 3.1.1. Design Concept

The vast variety of equipment and techniques applied to the filament-winding process necessitates a comprehensive analysis of the technology to justify the design configuration [36]. The common filament-wound structures used in aerospace programs are tanks, pressure vessels, motor casings, struts, and booms [37]. Thus, the selected design concept must be adapted to design the shells of revolution, preferably with a high length-to-diameter ratio and appropriate for laboratory applications or a single-unit production.

In a previously conducted study [38], all filament winders were divided into two groups, namely conventional and robotic, based on the type of the equipment. The former has a minimum required DOF, i.e., it is optimized for specific applications [36]. The application of an industrial robot is the typical characteristic of a robotic filament winding complex [39]. Technical and economical comparisons of the filament winders depend on a multitude of factors, with the most prominent being the size of the product being manufactured, configuration of the equipment, and the level of automation. In general, conventional FWM can accommodate mandrels with a high length-to-diameter ratio and robotic FWM are more adapted for small components with complex shapes [38]. However, the working envelope of robotic systems can be extended using additional linear axis. Despite the high market demand and competitiveness, which are among the principal merits of the conventional technology, the substitution of the rigid automation of the conventional winders with flexible automation typical for the robotic cells discernibly enhances the product value and market prospects [40,41]. However, robotic systems may require skilled personnel and increased maintenance costs. Apparently, the conventional equipment is more rational for the custom-made production of the components with a relatively simple axisymmetric shape and high length-to-diameter ratio.

Notwithstanding the heterogeneity in the layouts of the conventional filament winding equipment mentioned in the references [42,43], there are three archetypal basic configurations [44]: a lathe-type (helical) winder, a racetrack (in-plane or polar) winder, and a tumble winder. The racetrack (polar or in-plane) winders are effective for winding angles close to zero, but the length-to-diameter ratios of the wound parts are delimited to 1.8–2.0 to obviate the fiber slippage [43]. The tumble winder is efficient for the low-cost and high-speed manufacturing of spherical or “Dutch cheese”-shaped shells of revolution, but is not appropriate for winding hoop layers on cylindrical surfaces [45]. The configuration of a tumble winder suggested in reference [39] is capable of manufacturing long structures, but imposes demanding requirements on the rigidity of the mandrel holding structure. The lathe-type winders are the most prevalent and conventional machines with some limitations in the range of extremely small winding angles. Other disadvantages of the lathe-type machines include high delivery eye translations and accelerations in comparison with the racetrack or tumble winders. However, none of these drawbacks preclude the extensive use of the lathe-type configuration for manufacturing the axisymmetric aerospace structures mentioned above. Another cogent reason that renders a conventional lathe-type machine more appropriate for the applications presented here is the correlation of its cost with the number of DOF [36]. The sufficient number of DOF for the filament winder is determined by a number of independent parameters that describe the relative orientation between delivery eye and mandrel [39]. The greater the number of DOF the winder has, the more intricate the wound part and the control system are anticipated to be [46]. By the adoption of the conventional methods of linear algebra, it was shown that FWM must have at least six DOF [39]. However, for tow-winding, the sufficient number of DOF is four, as the twisting of the tow is of less importance. For the axisymmetric mandrel, some of the independent parameters might be constant. For instance, the distance between the surface of the mandrel and delivery tool might be constant for the helical tow-winding around the cylindrical mandrel. Thus, the number of sufficient DOF decreases to two. Components that are more complex than a cylinder can be wound with only two DOF [29]. It is worth noting that a low number of DOF requires a more elaborate calculation of the delivery eye trajectory and speed [31]. An additional DOF eliminates unrealistic translations in the winder components [32]. Consequently, the configuration with four DOF was selected for the filament winder under consideration.

From the three impregnation methods described in [47], only wet/dry winding has a widespread application for aerospace components. For occasional non-batch applications, the wet winding method is more advantageous when compared with the dry method for the following reasons [47,48]: (1) enhanced variety of fiber/resin combinations; (2) lower probability of fiber damage; (3) longer shelf life for resins, while prepregs must be stored in a freezer for a limited period; (4) room temperature cure; and (5) lower cost. Moreover, the wet winding equipment can be easily readjusted for prepreg winding.

The latter is considered available and straightforward from two roving impregnation systems, a dip-type and a drum-type bath system [49]. Notwithstanding the poor control over the fiber volume ratio that leads to an excess of resin, the drum-type bath systems have a wide application in industry. A doctor blade device may adjust the required resin-film thickness on the drum, restricting the amount of resin that can enter the polymer composite. Thus, the proposed low-cost technology for the irregular or laboratory production of the polymer composite shells of revolution with a high length-to-diameter ratio is based on a lathe-type filament winder with four DOF and a proper bath-type resin impregnation system. This low-cost concept is predicated on the extensive use of standard aluminum and steel profiles for the structural frames, off-the-shelf mechanical and electronic components, 3D-printed parts, obsolete personal computer and cheap software for the control system, and manual generation of the control codes supported by analytical equations. In addition, there are no complex adaptive tensioning systems. The pretension is provided by a simple tensioning mechanism, such as rotating scissor bars with manual adjustment [50]. The variation in fiber tension can be minimized by the appropriate feed-eye trajectory [31].

#### 3.1.2. Description of the Filament Winder

**Figure 5:**
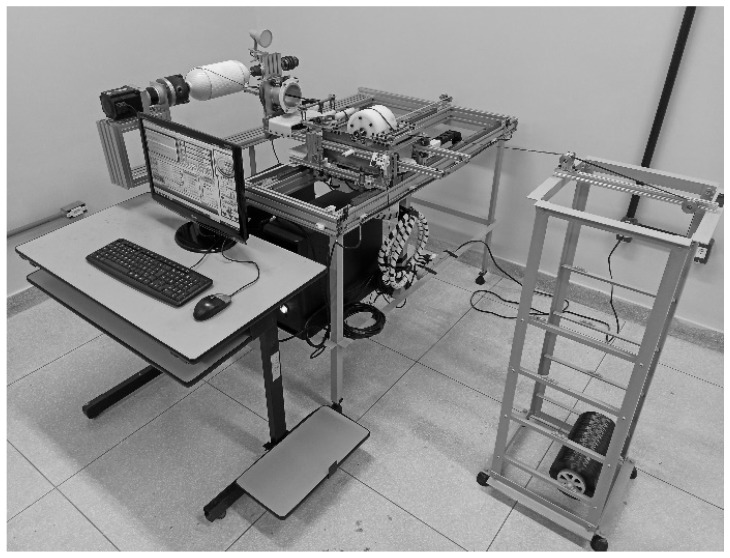
The devised low-cost filament winding system.

The system for the filament winding comprises the main units formed by the winder, the stationary creel, and the operator’s workplace (Figure 5). The stationary creel is a carbon steel shelf that holds a maximum of four fiber packages on a cardboard tube. The operator’s workplace is a desk equipped with a monitor, a keyboard, and a mouse. The winder was installed on a carbon steel stand holding cabinets for the control system, protected from resin leakage by a silicone layer.

**Figure 6:**
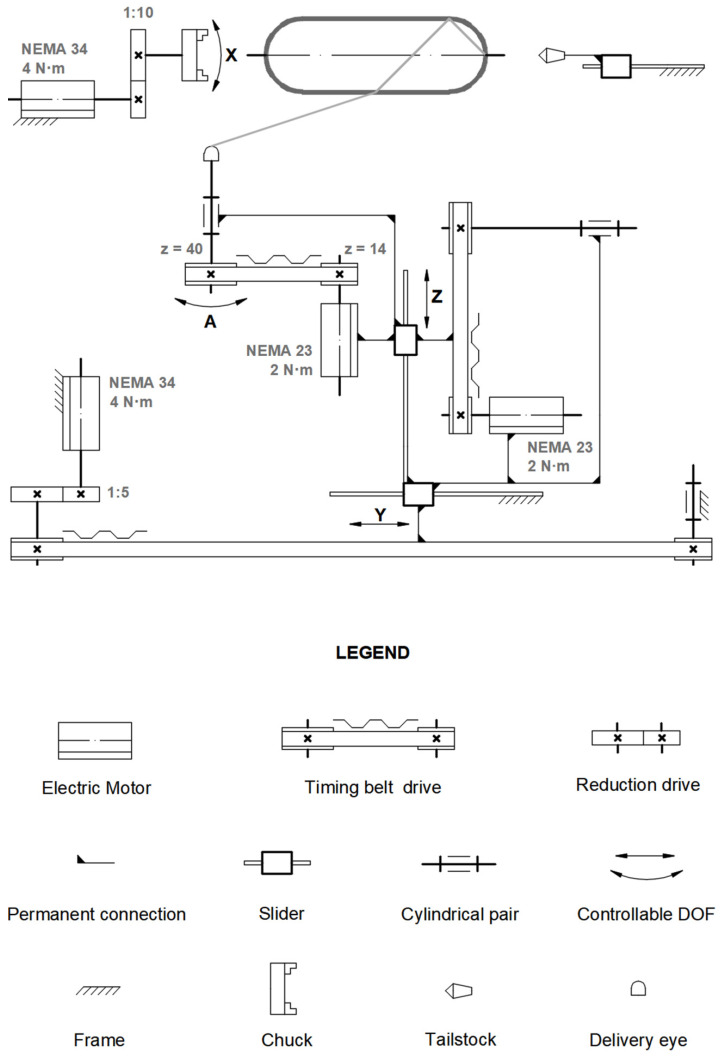
Kinematic diagram of the winder: N·m—unit for torque; NEMA—motor frame size; 1:5 and 1:10—reduction ratios; z—number of teeth.

There are four fully controlled axes in the filament winder (Figure 6): X is the rotation of the mandrel, Y is the linear translation of the carriage along the mandrel’s axis, Z is the linear translation of the delivery eye across the mandrel’s axis and A is the eye-rotation axis. A stepper motor with a frame size established by the National Electrical Manufacturers Association (NEMA) is the primary source of torque for all controllable axes. The torque from the stepper motors to the executive mechanisms is transferred through the timing belt drive (axes Y, Z, and A).

The planetary drives with reductions of 1:10 and 1:5 are installed between the stepper motor and the timing belt of axes X and Y, respectively.

**Figure 7:**
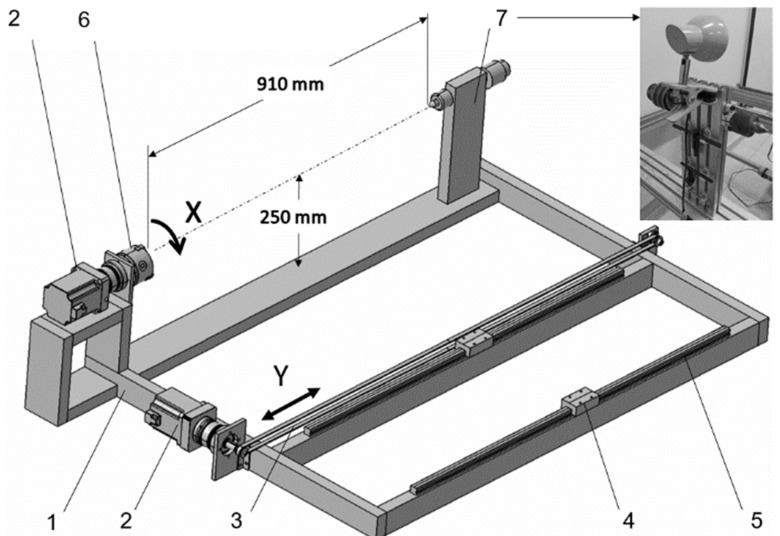
Assembly components of the filament winder frame (sensors are not shown): 1—aluminum V-slot profile; 2—stepper motor with planetary reduction drive; 3—timing belt; 4—slider block; 5—linear guide rail; 6—lathe chuck; 7—tailstock with tool set.

The winder consists of two primary assembly units: a frame and a carriage. The frame is made from a V-slot aluminum profile and holds the stepper motors of axes X and Y (Figure 7). The tailstock is equipped with both a rotating center and drill chuck to provide a variety of clamping methods. A pair of linear guides with slide blocks for installation of the carriage is also included.

**Figure 8:**
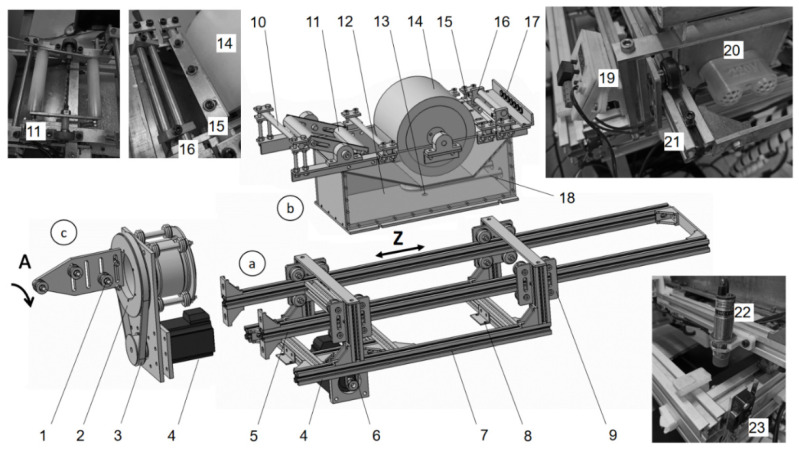
Assembly components of the carriage: (**a**) frame, (**b**) impregnator and (**c**) delivery head; 1—roller; 2—hollow pulley; 3—closed timing belt; 4—stepper motor; 5—brace; 6—timing belt; 7—frame; 8—bracket; 9—gantry plate with mini wheels; 10—static bar; 11—tension compensator; 12—water basin; 13—drain; 14—impregnation drum; 15—doctor blade; 16—rotating scissor bars; 17—guide; 18—heating element; 19—thermostat; 20—terminal for the heating element; 21—fixture of the timing belt at the end of the brace; 22—proximity sensor Z; 23—limit switch Y.

The carriage encompasses three subassemblies (Figure 8): the carriage frame, the impregnator, and the delivery head. The carriage frame is made from a V-slot aluminum profile (Figure 8a). Two braces are installed in a gantry plate between the mini wheels. The timing belt, whose ends are fixed to the extremities of the braces, provides linear translation Z of the braces. The middle portion of the belt is pulled over the GT2 timing pulley, which is driven directly by the stepper motor. The impregnator (Figure 8b) is installed between the carriage braces and consists of a heated resin bath with an impregnation drum, rotating scissor bars with manual adjustment, a couple of guides, and a tension compensator with a torsion spring. The tension compensator works similarly to a rotating scissor bar and the distance between the bars can also be adjusted. The hollowed drum is made from high density polyethylene to facilitate rotation. The guides are made from stainless steel or polyoxymethylene (POM). The delivery head (Figure 8c) is responsible for the fourth controllable DOF and consists of an aluminum tube supported by a set of mini wheels. The tube holds the delivery eye with a couple of rollers made from POM.

#### 3.1.3. Control System

The control system of the filament winder is based on commercial software ArtSoft Mach3, a widely used solution for custom-made CNC machines [51]. The Mach3 software, in combination with a breakout board, virtually transforms a PC into a CNC machine controller.

**Figure 9:**
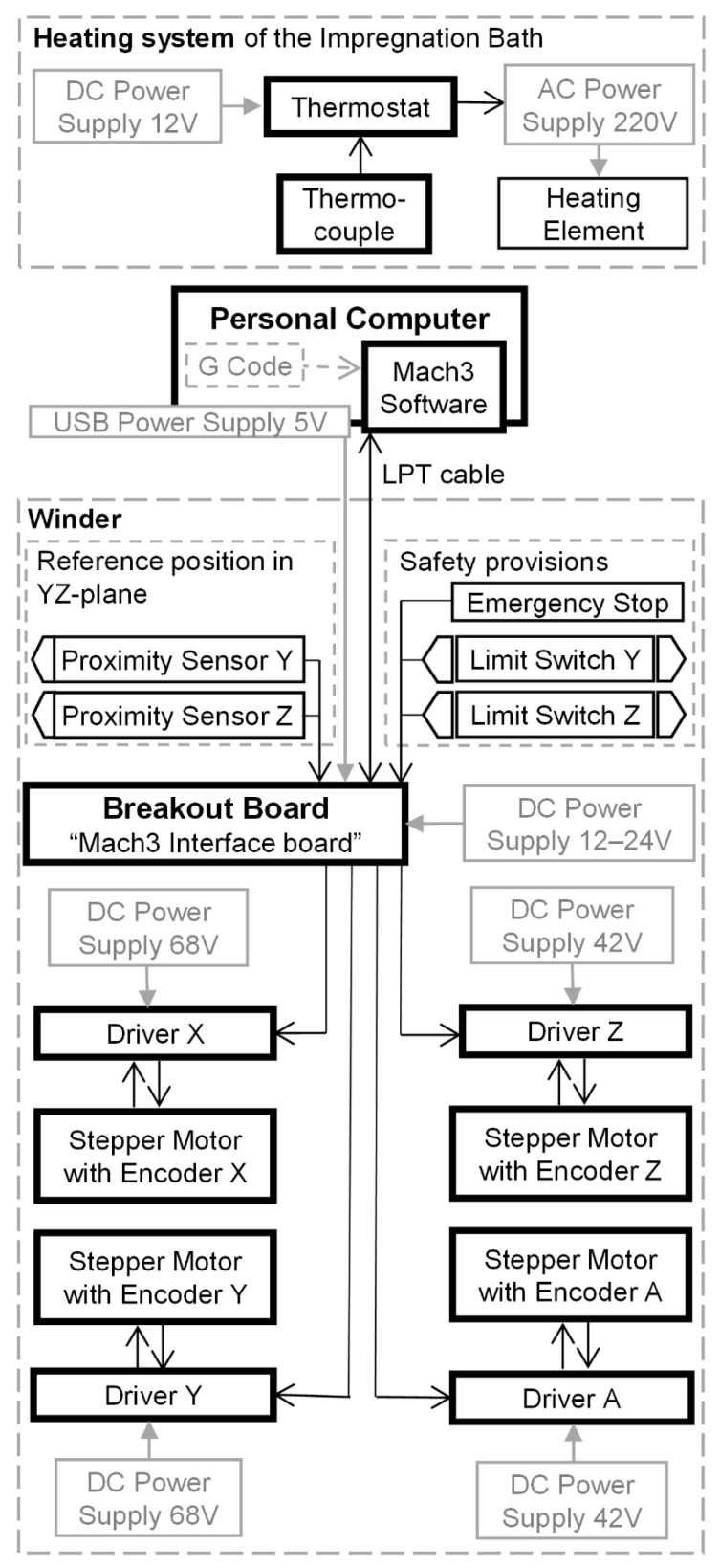
Control system diagram.

The breakout board was incorporated to translate signals from the PC to the winder’s components (drivers, switches, and sensors) and vice versa (Figure 9). The low-cost isolating breakout board “Mach3 Interface Board,” adopted for the control system, also functions as circuit protection. The requirement of an auxiliary 12–24 V power supply for the switch control can be confirmed as the primary disadvantage of the board.

A low-performance PC (1 GHz processor) with an obsolete version of Microsoft Windows (2000, XP, Vista) can be employed for a direct connection from the PC’s motherboard to the “Mach3 Interface Board” through a parallel port [52]. This approach considerably reduces the cost of the control system. The stepper motors with encoders are used to enhance the accuracy of tow deposition. Contact-limit switches are used to prevent the Y and Z linear axes from causing damage to the structure of the winder. The winder is equipped with relocatable proximity sensors, whose primary function is to establish a home position or a reference position (origin of the Y and Z coordinates). The heating control of the impregnation bath is separated from the control system. It is equipped with a simple thermostat that maintains a predetermined temperature of the resin in the impregnation bath.

### 3.2. Trajectory of the Delivery Eye

To place the tow along the geodesic path when the distance between the delivery eye and the mandrel surface is not zero, equations for the coordinates of the delivery-eye trajectory are indispensable.

**Figure 10:**
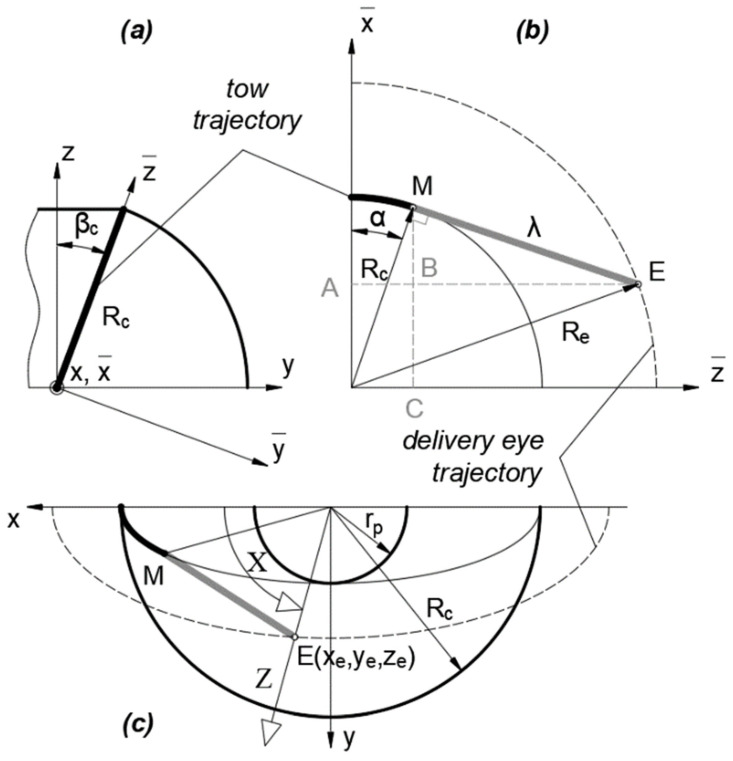
Delivery eye trajectory for the spherical dome: (**a**)—*yz*-plane; (**b**)—$\bar{x}\bar{z}$-plane; (**c**)—*xy*-plane.

The tow runs over a large circle of the sphere [34], thus the trajectory of the tow is a circle that lies in a plane. The coordinate system of the plane $\bar{x}\bar{y}\bar{z}$ is formed by an elemental rotation of the mandrel’s coordinate system *xyz* about axis *x* by an angle $\beta_{c}$ (Figure 10a). For simplicity, it is considered that the trajectory of the delivery eye is also a circle (Figure 10b), and therefore, the distance *λ* between the delivery eye *E* and the mandrel surface *M* is constant.

The closed-line segment *EM* must be tangential to the surface of the dome. It is calculated through the radius *R_e_*, defined by the operator:

(12) $$\lambda =\sqrt{R_{e}^{2}-R_{c}^{2}.}$$

The coordinates of the delivery eye in the coordinate system $\bar{x}\bar{y}\bar{z}$ are defined through the variable angle *α* depicted in Figure 10b:

(13) $${\bar{x}}_{e}=MC-MB=R_{c}\cos\alpha -\lambda \sin\alpha ,\;{\bar{y}}_{e}=0,\;{\bar{z}}_{e}=AB-BE=R_{c}\sin\alpha -\lambda \cos\alpha .$$

The coordinates of the delivery eye in the mandrel’s coordinate system xyz are defined by the following well-known equations of linear algebra:

(14) $$x_{e}={\bar{x}}_{e},\;y_{e}={\bar{y}}_{e}\cos\beta_{c}-{\bar{z}}_{e}\sin\beta_{c},\;z_{e}={\bar{y}}_{e}\sin\beta_{c}+{\bar{z}}_{e}\cos\beta_{c}.$$

Substituting Equations (13) into (14) provides

(15) $$x_{e}=R_{c}\cos\alpha -\lambda \sin\alpha ,\;y_{e}=-\frac{r_{p}}{R_{c}}\left(R_{c}\sin\alpha +\lambda \cos\alpha \right),\;z_{e}=\frac{\sqrt{R_{c}{}^{2}-r_{p}{}^{2}}}{R_{c}}\left(R_{c}\sin\alpha +\lambda \cos\alpha \right).$$

The coordinates of the delivery eye are obtained through controllable DOF (i.e., in the coordinate system of the winder), which are determined from Figure 10c in terms of the coordinates in the mandrel’s coordinate system *xyz*.

(16) $$X=\tan^{-1}\frac{y_{e}}{x_{e}},\;Y=z_{e},\;Z=\sqrt{x_{e}{}^{2}+y_{e}{}^{2}}.$$

Finally, substituting Equations (15) into (16) provides the formulas for the delivery eye trajectory as a function of *α*

(17) $$X=\tan^{-1}\left[-\frac{r_{p}\left(R_{c}\sin\alpha +\lambda \cos\alpha \right)}{R_{c}\left(R_{c}\cos\alpha -\lambda \sin\alpha \right)}\right],\;Y=\frac{\sqrt{R_{c}{}^{2}-r_{p}{}^{2}}}{R_{c}}\left(R_{c}\sin\alpha +\lambda \cos\alpha \right),\;Z={\left({\left(R_{c}\cos\alpha -\lambda \sin\alpha \right)}^{2}+{\left(\frac{r_{p}}{R_{c}}\right)}^{2}{\left(R_{c}\sin\alpha +\lambda \cos\alpha \right)}^{2}\right)}^{1/2}.$$

**Figure 11:**
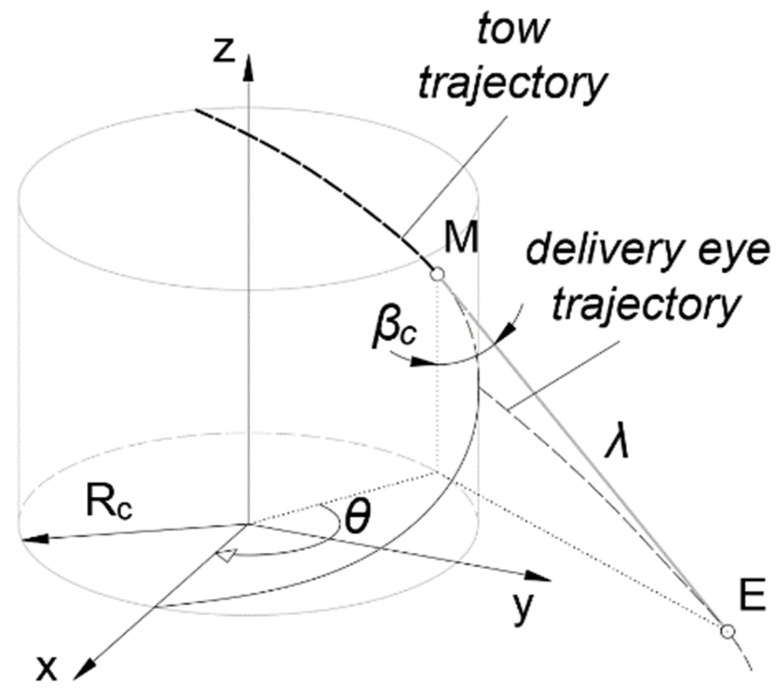
Delivery eye trajectory for the cylinder.

An involute screw surface analytically described in a Cartesian coordinate system [53] provided the coordinates of the delivery eye trajectory for the cylindrical part of the mandrel (Figure 11):

(18) $$x_{i}=R_{c}\cos\theta +\lambda \sin\beta_{c}\sin\theta ,\;y_{i}=R_{c}\sin\theta -\lambda \sin\beta_{c}\cos\theta ,\;z_{i}=R_{c}\theta \cot\beta_{c}-\lambda \cos\beta_{c}.$$

Here, angle θ is a function of the axial coordinate *z*:

(19) $$\theta =\frac{z\tan\beta_{c}}{R_{c}}.$$

The delivery eye trajectory is given through controllable DOF of the winder as follows:

(20) $$X=\frac{z\tan\beta_{c}}{R_{c}},\;Y=z,\;Z={\left({\left(R_{c}\cos\theta +\lambda \sin\beta_{c}\sin\theta \right)}^{2}+{\left(R_{c}\sin\theta -\lambda \sin\beta_{c}\cos\theta \right)}^{2}\right)}^{1/2}$$

The angle of the eye rotation axis *A* depends on the winding angle, such that the axis of the roller must be perpendicular to the direction of the tow:

(21) $$A=\frac{\pi }{2}-\beta$$

Here, *β* is calculated by Equation (3) for the hemispherical dome and by Equation (1) for the cylinder.

### 3.3. Validation of the Analytical Solution for the Winding Trajectory of the FWM

The length of the cylindrical part of the mandrel *L_c_* = 84.1 mm is chosen in such a manner that the imprinted grooves are continuous along the geodesic path (see Figure 2) and form a closed loop.

**Figure 12:**
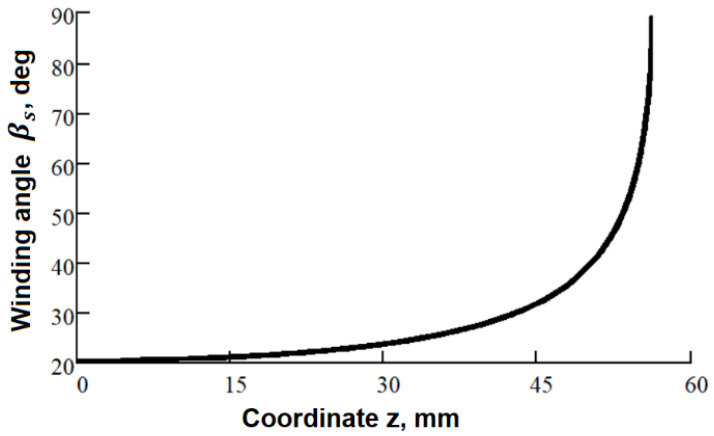
Winding angle on the spherical dome.

**Figure 13:**
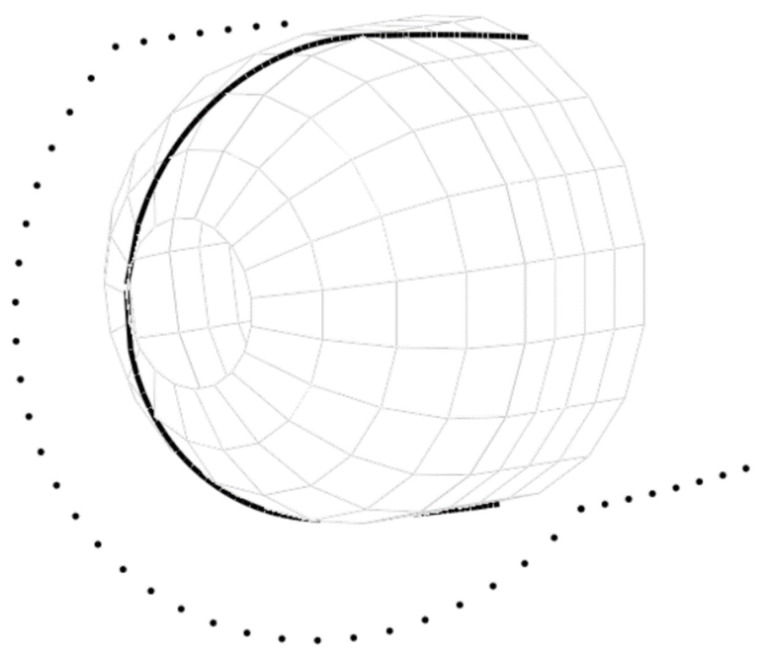
Calculated trajectories of the delivery eye and the tow depicted for half of the mandrel (*λ* = constant).

**Figure 14:**
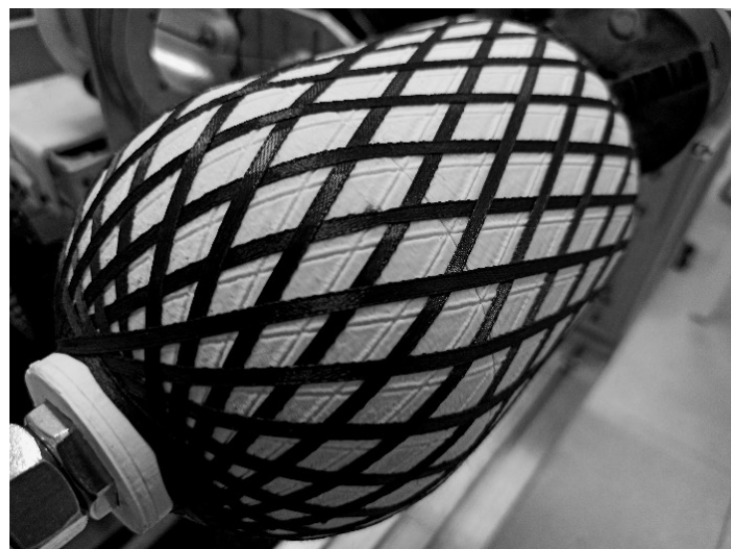
Wound pattern on the surface of the mandrel with the imprinted geodesic path.

The winding parameters (Table 3 and Figure 12) yield the same pattern on the surface of the mandrel for both the tow and the grooves. The trajectories of the delivery eye and the tow calculated for the constant distance *λ* = 67 mm by Equations (17) and (20) are illustrated in Figure 13. The wound pattern obtained by the calculated trajectories (the generated G-code is given in Appendix A) corresponds to the pattern of the imprinted grooves (Figure 14), which, in turn, substantiates the reliability and effectiveness of the suggested equations.

As the geodesic trajectory has been calculated for a filament with an infinitesimally small width, a strip with a width of 3.5 mm partially covers the surface of the flange. Overlap can be eliminated by decreasing the opening radius in the calculations of the geodesic path or can be regulated with a slight displacement of the initial position of the delivery eye out of the mandrel. However, deviations from the geodesic path are inevitable in the latter case.

### 3.4. Characterization of the Casings

The winding parameters used for the manufacturing of the casings are the same for all of the double helical plies in the layup (Table 4 and Figure 12). The winding pitch for the 90° ply is 4 mm.

**Figure 15:**
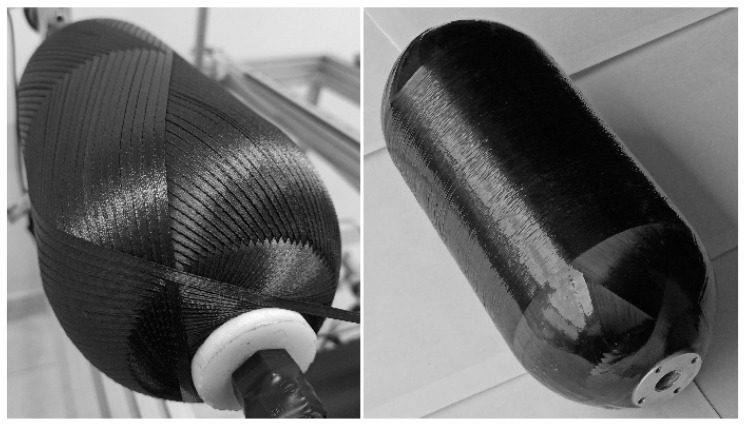
Wound pattern of the double helical layer verified with synthetic strip (**left**) and the carbon/epoxy casing LY5052 [±20.5_3_/90] after curing (**right**).

Before winding the casings with carbon filament impregnated by two types of the epoxy resin, the calculated trajectory of the delivery eye was already successfully verified for the double angle-ply layer with use of the synthetic strip (Figure 15). The winding of one double angle-ply layer required approximately 16 min at the maximum speed established in the G-code, which is equal to 6000 mm/min for the linear translations (Z and Y axes) and 6000 degrees/min for the rotational motion (X and A axes).

**Figure 16:**
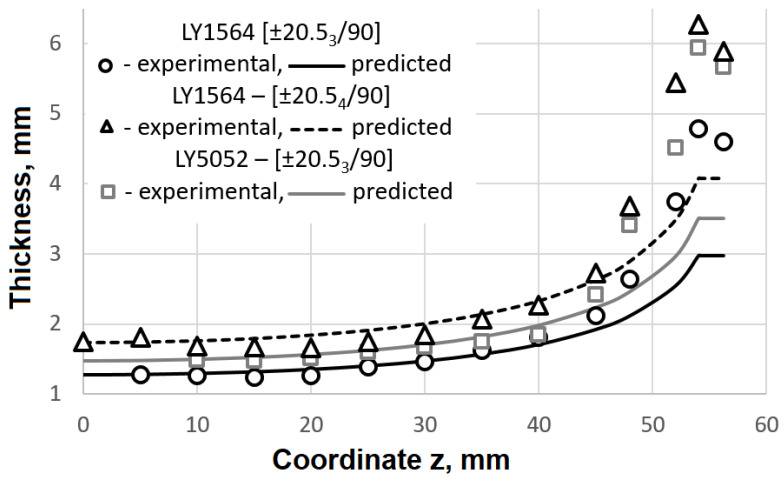
Thickness distribution in the dome of the wound casings.

**Figure 17:**
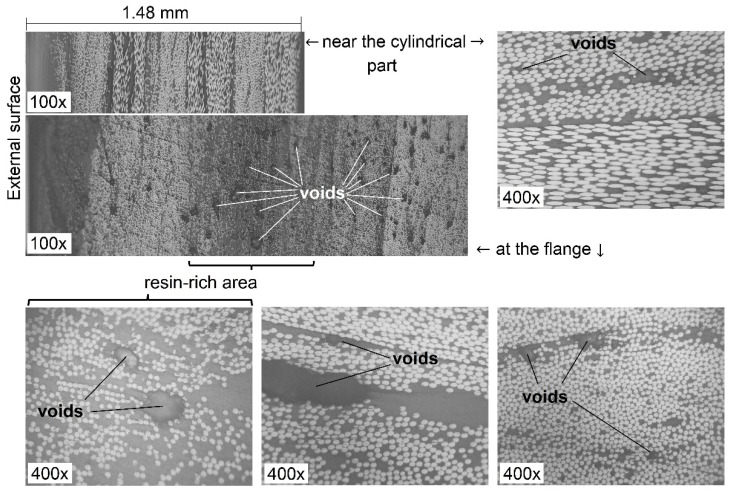
Micrographs at two opposite extremities of the dome of the casing LY5052 [±20.5_3_/90]: voids.

**Figure 18:**
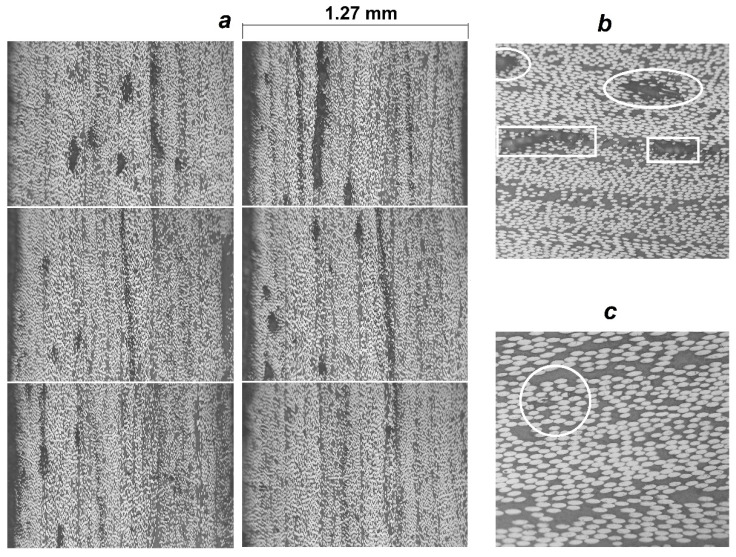
The selected micrographs 100× (**a**) of the dome near the cylindrical part of the casing LY1564 [±20.5_3_/90] with different content of the voids: (**b**) inter-ply voids in rectangular marker and intra-ply voids in elliptical marker (200×); (**c**) intra-ply void between broken fibers (400×).

The thicknesses measured along the dome wall of the wound casings with different resin compositions and stacking sequences are in good agreement with the distribution predicted by the flat solution, except for the portion near the flange (Figure 16), as a result of the varying compaction performance of the composite material at the dome. Notwithstanding the significant amount of resin drips from the casing surface before the curing is completed, a great volume of resin is trapped inside the layer (Figure 17). At the flange, there is a huge zone covered in resin and there are multiple pores. In the cylindrical part, the trapped resin is between the plies and there are few pores. The most porous casing is made from composition LY1564 (Figure 18). The size and the distribution of voids can be attributed to the absence of an adaptive tensioner of the tow in the developed FWM and excessive resin content inherent to the drum-type bath impregnation system. In the latter case, the voids originate from entrapped air bubbles or from more complex local curing effects described in the literature [54].

The evaluated fiber volume ratio is in the range of 52–55% for the casing LY5052 [±20.5_3_/90] and in the range of 50–56% for the casing LY1564 [±20.5_3_/90]. These data are given for the near cylindrical parts of the casing, excluding the layer of the pure resin on the external surface shown in Figure 17.

Although the winder can lay the fiber on the surface of the mandrel straight along the geodesic path (Figure 14), there are compromises to be made in using the low-cost design solutions. For the given setup, the fiber volume ratio decreases by up to 50–52% in the cylindrical casing, depending on the resin type. According to [55], the change in the fiber volume fraction from 50% to 65% improves the strength of the composite by at least 10%.

## 4. Conclusions

The presented work validates the possibility of manufacturing axisymmetric composite structures, such as pressure vessels, with low-cost filament winding equipment. The cost-effective system relies on off-the-shelf components, an obsolete computer, and an affordable control system. The manually generated G-codes using the derived analytical equations for the delivery eye trajectory further reduce the cost of the system. The equations are obtained by referring only to analytic geometry and do not demand additional efforts or specific skills from an FWM operator. The preliminary results of manufacturing trials showed that the winder lays the fiber straight along the geodesic trajectory. Despite this fact, there might be compromises in using low-cost design solutions. Therefore, further works will investigate the performance of wound composite structures and the winding accuracy of other axisymmetric and non-axisymmetric shapes. Consequently, the suggested low-cost equipment provides small research teams with the possibility to carry out their projects, at least in the initial development stages.

## Figures and Tables

**Table 1:** Properties of the carbon fiber tow ^1^.

Parameter	Minimal	Nominal	Maximal
$\rho_{f}$, tex	720	800	880
ρ, g/cm^3^	1.77	1.80	1.83
$F_{1f}$, MPa	4050	4500	4950
$E_{1f}$, GPa	228	240	252

^1^ as affirmed by local supplier Texiglass.

**Table 2:** Cure schedules for three types of resin-impregnated tows.

Designation	Resin Composition	Initial Viscosity at 25 °C ^1^, mPa·s	Cure Schedule
LY1564	Araldite LY1564/XB3473	1000–1200	30 min at 130 °C + 12 h at 160 °C
LY5052	Araldite LY5052/Aradur TM5052	600–700 (to 1500 after 56–60 min)	24 h at 25 °C + 15 h at 60 °C

^1^ manufacturer’s data.

**Table 3:** The winding parameters for the mandrel with imprinted geodesic path, in degrees.

*β_c_*	Φ*_c_*	Φ*_s_*	Φ_1_	Φ*_p_*	Φ*_f_*	Φ*_w_*
20.5	15	90	420	0	0	15

**Table 4:** The winding parameters for the mandrel with imprinted geodesic path, in degrees.

*β_c_*	Φ*_c_*	Φ*_s_*	Φ_1_	Φ*_p_*	Φ*_f_*	Φ*_w_*
20.5	57.1	90	474.2	120	2.91	4.08

**Figure A1:**
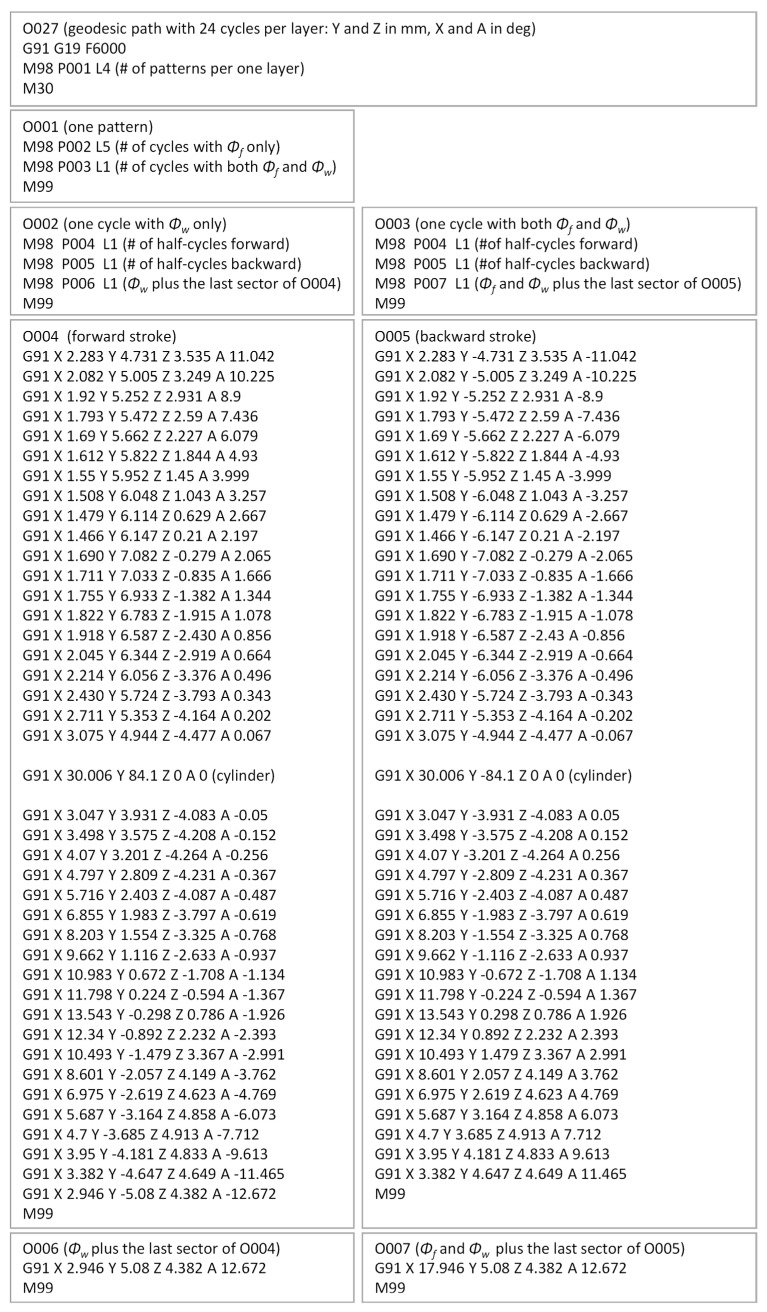
G-code for the winding trajectory on the mandrel with an imprinted geodesic path (*R_c_* = 60 mm, *r_p_* = 21 mm, and *L_c_* = 84.1 mm).

## Data Availability

The data presented in this study are available on request from the corresponding author.

## Abbreviations

E	position of the delivery eye on its trajectory;
$E_{1f}$	tensile modulus of the fiber;
$F_{1f}$	tensile strength of the fiber;
$L_{c}$	length of the cylindrical part;
M	point of tangency of the fiber to the surface of mandrel;
$R_{c}$	radius of the cylindrical casing and hemispherical dome;
$R_{e}$	radius of the circular trajectory of the delivery eye;
$r_{p}$	radius of polar opening;
$t_{d}\left(z\right)$	thickness distribution in the dome (excluding the portion near the opening) as a function of the z-coordinate;
$t_{do}$	thickness in the dome near the opening;
$z_{p}$	z-coordinate of the polar opening;
α	angle between axis $\bar{x}$ and radius vector to the point M in the plane $\bar{x}\bar{y}\bar{z}$;
β	winding angle;
$\beta_{c}$	winding angle on the cylindrical part;
$\beta_{s}\left(z\right)$	winding angle on the hemispherical dome as a function of the z-coordinate;
η	initial viscosity, mPa·s
λ	distance between the delivery eye and point of tangency of the fiber to the surface of mandrel;
$\rho_{f}$	linear mass density of the fibers;
Φ	turn-around angle (an angle of mandrel rotation to lay the tow along its predetermined trajectory);
$\Phi_{c}$	turn-around angle for the cylindrical part;
$\Phi_{s}\left(z\right)$	turn-around angle for the hemispherical dome as a function of the z-coordinate;
$\Phi_{1}$	turn-around angle for one winding cycle;
$\Phi_{p}^{*}$	calculated angular pitch of winding;
$\Phi_{p}$	accepted angular pitch of winding;
$\Phi_{f}$	turn-around angle per one flange;
$\Phi_{w}\;$	turn-around angle for the width of the tow.
CNC	computer numeric control
DOF	degrees of freedom
FWM	filament winding machine
LVDT	linear variable differential transformer
NEMA	National Electrical Manufacturers Association
POM	polyoxymethylene
PC	personal computer
